# A strategic approach to regulating new antimicrobials

**DOI:** 10.7189/jogh.15.03033

**Published:** 2025-08-18

**Authors:** Shilpi Malhotra, Nitin Bansal, V Ramasubramanian, Veeraraghavan Balaji, Leena Menghaney, Kamini Walia

**Affiliations:** 1 Division of Descriptive Research, Indian Council of Medical Research, New Delhi, India; 2 Department of Infectious Diseases, Apollo Hospitals, Chennai, India; 3 Department of Clinical Microbiology, Christian Medical College, Vellore, India; 4 Pharmaceuticals and Access, Médecins Sans Frontières, Delhi, India

## Abstract

Antimicrobial resistance (AMR) is a significant threat to healthcare in India, which is experiencing increasing resistance to critical antibiotics, including last-resort treatments. Emerging data indicate that resistance to last resort life-saving drugs continues to rise, limiting treatment options for drug-resistant infections. While the introduction of new antimicrobials offers hope, historical patterns suggest that resistance may develop rapidly. Enforcement challenges of regulatory initiatives, such as Schedule H1 restrictions in India, underscore the need for a national-level strategic approach when introducing new antimicrobials in the future. We propose a regulatory and access framework designed to preserve and increase the longevity of new antimicrobials in India. Key recommendations include restricting their availability to formularies of hospitals implementing antimicrobial stewardship and infection prevention and control standards, and mandating AMR surveillance reporting. We also examine stakeholders’ perspectives on policy measures necessary for the responsible introduction of these drugs.

Antimicrobial resistance (AMR) is a major challenge for healthcare systems worldwide, including India, where the emergence of resistance to antibiotics, including last resort antibiotics in India, is alarming. Data from the Indian Council of Medical Research (ICMR) AMR surveillance network highlights rising antibiotic resistance rates, with *Klebsiella pneumoniae* showing 62.3% resistance to carbapenem (specifically meropenem). The susceptibility of *Escherichia coli* to imipenem has declined from 81% in 2017 to 63% in 2023, while its sensitivity to piperacillin-tazobactam dropped from 56.8% to 42.4% during the same period [1]. *Klebsiella pneumoniae* isolates also exhibited low susceptibility to piperacillin-tazobactam, at just 26.5% in 2023 [1]. High resistance to carbapenems and piperacillin-tazobactam has significantly limited treatment options for infections caused by pathogens resistant to multiple drugs. Medical practitioners in India are therefore compelled to prescribe polymyxins, such as colistin, as a last-resort treatment [2]. Yet an uptick in colistin prescriptions is contributing to the rise in resistance to this antibiotic as well [3]. Over the past decade, all newly introduced antimicrobials failed to demonstrate improved effectiveness against multidrug-resistant gram-negative pathogens, which are major contributors to the antimicrobial resistance burden in India [4]. Therefore, given the rise in resistance of pathogens to available drugs, new treatment options are urgently needed. Several new antimicrobials, such as plazomicin, cefiderocol, and novel β-lactam combinations (which are awaiting registration in India), may offer a promising solution.

Das and colleagues showed that resistance tends to develop shortly after the introduction of any new antibiotic [5]. For example, ceftazidime avibactam was introduced in India in 2018, with generic formulations becoming available from 2023 onwards. Resistance to ceftazidime-avibactam has been observed across multiple gram-negative pathogens, based on unpublished data from the ICMR. Among *Escherichia coli* isolates, 189 out of 503 (37.6%) were resistant. Resistance was notably higher in *Klebsiella pneumoniae*, with 388 out of 518 isolates (74.9%) showing non-susceptibility. *Pseudomonas aeruginosa* also demonstrated significant resistance, with 98 out of 180 isolates (54.4%) found to be resistant to ceftazidime-avibactam. Expanded availability of new antimicrobials in Indian markets could lead to their overuse and to the rapid development of resistance [6]. A national-level policy that regulates the use of new antimicrobials could ensure that the drugs remain effective longer.

## EXISTING REGULATORY FRAMEWORKS IN INDIA

Since the introduction of the National Policy for Containment of Antimicrobial Resistance in India in 2011, the Indian Government has implemented various regulatory strategies to curb the inappropriate use of antimicrobials. These measures include the introduction of Schedule H1 to restrict over-the-counter sales of antimicrobials, the launch of the Red Line Campaign to raise public awareness, and periodic revision of the National List of Essential Medicines [7]. However, these initiatives had limited success, likely due to implementation challenges [8–10].

A more robust example of regulatory stewardship for new antimicrobials, with an intention to increase the longevity of drugs while ensuring access for patients in India can be seen in the case of the tuberculosis (TB) programme. The rollout of new TB drugs for drug-resistant TB – bedaquiline, delamanid, and pretomanid – is only allowed through healthcare facilities registered as DOTS-Plus centres in an online portal, which in turn managed TB notification, treatment, and financial aid for nutrition for patients [11]. Diagnostic quality is maintained by involving public and private laboratories accredited for culture and drug susceptibility testing. Although restricting access to new TB drugs and ensuring their judicious use caused short-term challenges, it has resulted in long-term benefits in managing multidrug-resistant TB patients [12,13].

## LESSONS FROM OTHER COUNTRIES

The importance of regulating the use of antimicrobials has also been documented in other countries. By combining real-time surveillance, professional and public engagement, and credible national targets, Sweden’s Strama programme has enabled sustainable behaviour change through a bottom-up model that directly linked prescribers with policymakers [14]. Australia’s National Antimicrobial Prescribing Survey has emphasised the role of systematic auditing in improving prescribing practices by providing detailed feedback to healthcare facilities, thus facilitating data-driven interventions and informed national policy [15].

Brazil’s Health Regulatory Agency has developed guidelines to encourage the implementation of antimicrobial stewardship (AMS) programmes in hospitals, focussing on optimising antimicrobial use based on local needs and resources [16]. The country also strengthened its surveillance systems, such as BR-GLASS, to monitor antimicrobial resistance patterns and inform policy decisions and treatment guidelines [17].

China’s Administrative Regulations for Clinical Use of Antibacterial Agents 2012 classified antibiotics into non-restricted, restricted, and special-grade categories, linking prescribing privileges to physicians’ qualifications and institutional levels [18]. Physicians and pharmacists are required to undergo specific training in antibiotic prescription, while hospitals’ stewardship committees monitor antibiotic use and penalise non-compliance, including revoking prescribing rights [19].

All these initiatives highlight the importance of coordinated, multi-sectoral strategies in combating antimicrobial resistance. By learning from global best practices and experience from the TB programme within the country, India can enhance its efforts to introduce new antimicrobials responsibly and curb the rise of antimicrobial resistance.

## A REGULATORY APPROACH TO PREVENT MISUSE

Effective antimicrobial stewardship requires ensuring timely and equitable access to life-saving antimicrobials and preventing their misuse. India has prioritised AMS, and all medical colleges are now required to have a functional AMS committee [20]. The ICMR’s AMS programme in selected hospitals has seen reasonable success [21]. However, change through AMS is tardy, and ensuring sustainability remains a practical challenge [22]. Proactive regulatory measures can complement the ongoing efforts in ensuring the long-term effectiveness of life-saving antimicrobials.

The ICMR convened a meeting of experts, clinicians, microbiologists, and regulators to strategise a regulatory mechanism for new antimicrobials yet to be introduced in the Indian market. The expert group’s recommendations are as follows:

1. New antimicrobials in the Watch and Reserve category meant for sick and hospitalised patients should not be allowed to be dispensed from retail pharmacies. Their availability should be restricted only to hospital formularies. Hospitals planning to procure and prescribe these new antimicrobials should:

- provide documentary evidence of compliance with pre-mandated standards for infection prevention and control (IPC) and AMS certification from a nationally recognised body.
- periodically submit AMR surveillance data to a designated national level platform and declare it on the hospital website; here, non-compliance should invite punitive measures, such as temporary suspension of access to restricted antimicrobials;
- have a functional AMS committee to undertake review and audit of prescriptions and a commitment to share data with the national regulatory and other agencies;
- have an in-house pharmacy, a clinical pharmacist, and diagnostic capacity for rationalising prescriptions and a mechanism to monitor the antibiotic prescriptions down to the individual patient.

2. To strengthen the regulatory oversight, a centralised, risk-based post-marketing surveillance system should be established, with mandatory reporting for manufacturers, regarding the sale of these antimicrobials.

**Figure 1:**
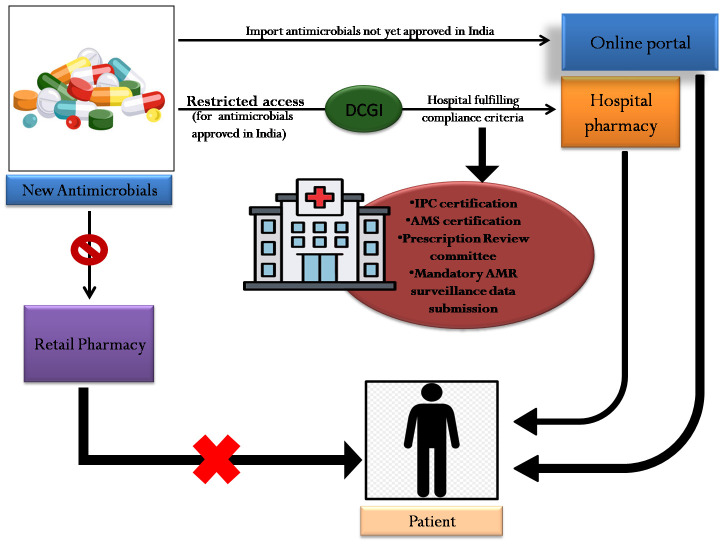
Suggested pathway for introduction of new antimicrobials.

Given the unique challenges of India’s healthcare system, it is crucial that the proposed strategy provides necessary access to patients in both public and private hospitals which may not have the required AMS framework in place to allow access to these antimicrobials. A phased regulatory approach can be followed to introduce new antimicrobials, while allowing for capacity building and ensuring sustained implementation of antimicrobial stewardship practices across the healthcare system. Tertiary care hospitals can actively mentor and guide smaller healthcare facilities, including district hospitals, community health centres, and nursing homes, using tools such as telemedicine-based pre-approval systems for smaller nursing homes, supported by a regulated supply of high-end antimicrobials from tertiary hospitals. The inclusion of high-priority antimicrobials in public procurement and supply schemes can be encouraged, particularly for government facilities and private sector facilities willing to engage in such a mechanism, to expand access to newer antimicrobials where they are most needed [23]. Until the drug receives approval from the regulator, CDSCO’s SUGAM portal can be utilised to import drugs for patients who need them [24]. The suggested pathway for the introduction of new antimicrobials in India is presented in **Figure 1**.

## ADVOCACY FOR RATIONAL ANTIMICROBIAL USE: EXPERTS’ PERSPECTIVES

The success of strategies aimed at regulating antimicrobial use heavily relies on the attitudes of stakeholders involved in the prescribing process. The ICMR surveyed clinicians and public health experts on prescribers’ perceptions of the factors driving misuse and the need to safeguard new broad-spectrum antimicrobials meant for treating serious infections. A questionnaire (**Online Supplementary Document**) was administered to 35 infectious diseases physicians, 19 clinical pharmacists, 16 clinical microbiologists, 4 public health specialists, and 29 clinicians, and purposive snowball sampling was used for selecting the survey participants. Most of the experts (73.1%) identified diagnostic uncertainty as the key factor responsible for unnecessary antimicrobial usage, followed by lack of availability of antibiograms (51.9%) (Figure S1 in the **Online Supplementary Document**). Most experts (86.5%) relied on national/international guidelines for choosing antibiotics, 71.2% referred to local hospital guidelines, and 55.8% sought advice from senior colleagues, infectious diseases physicians, microbiologists or clinical pharmacists. Only 1.9% experts believed that antibiotic prescriptions are based on endorsements from representatives of pharmaceutical companies (Figure S2 in the **Online Supplementary Document**). Moreover, since most of the survey participants were from tertiary hospitals with training in AMS, preserving the patient-doctor relationship was not a major driver for clinicians. Similarly, the participants showed low reliance on medical representatives, contrary to previously reported studies from India [25].

Nearly all participants (98.1%) advocated for a new antimicrobial regulatory policy to prevent misuse, while 55.8% recommended that new antimicrobials be available only in hospital formularies (Figure S3 in the **Online Supplementary Document****)**. Participants supported the idea of qualifying criteria for hospitals to introduce new antimicrobials. A key limitation of this study is the narrow representation of stakeholders, as the survey primarily involved experts from tertiary care hospitals, excluding general practitioners, rural healthcare providers, and retail pharmacists. Including a broader range of stakeholders in future studies would provide more comprehensive insights to inform policy.

## CONCLUSIONS

Traditionally, antimicrobials are sold in India through retail markets and hospital formularies. While this policy promotes access and favours the autonomy of the clinicians, it does little to check misuse. Since the existing treatment options for multidrug-resistant infections are rapidly depleting, it is necessary to explore all interventions that can help prevent the misuse of newer antibiotics. The ICMR’s recommendations on restricting access to new antimicrobials provide a roadmap to safeguard these drugs and establish a pathway for their ‘conservation’. Recommended mandatory standards, such as IPC and AMS certification from a recognised national body for hospitals and mandatory submission of AMR surveillance data, would serve the country’s interest towards improving patient care, as more hospitals would strive for accreditations and also become cognisant of their own AMR data. This guidance also aligns with our findings, suggesting regulatory control for new antimicrobials by limiting their availability to hospital formularies to prevent their misuse.

## Additional material


Online Supplementary Document
