# Supplementary material for: A strategic approach to regulating new antimicrobials
Source: J Glob Health. 2025 Aug 18;15:03033. doi: 10.7189/jogh.15.03033 (PMC12363153; doi:10.7189/jogh.15.03033)
Supplement: Online Supplementary Document [file jogh-15-03033-s001.pdf]

Supplement to: Malhotra S, Bansal N, Ramasubramanian V, Balaji V, Menghaney L, Walia K. A strategic approach to regulate new antimicrobials. J Glob Health. 2025;15:03033.

Supplementary information:

The following questionnaire was administered to the study participants:

I've invited you to fill out a form:

Identifying a pathway for introduction of new antimicrobials in India: a survey of physicians perspective by Indian Council of Medical Research

Antimicrobial resistance (AMR) is a major challenge faced by the healthcare systems worldwide including India. Emergence of resistance to newer antimicrobial agents is threatening the availability of antibiotics of last resort to tackle MDR pathogens. This is especially true for patients suffering from serious bacterial infections where the treatment options are limited by increasing levels of resistance to drugs like carbapenems, piperacillin-tazobactam and other such broad spectrum drugs. In India, most antibiotics are placed under Schedule H1 and can be dispensed by the registered pharmacists only on the prescription of a Medical practitioner. There have been few examples within the country where the government of India introduced a policy of restricted access to prevent misuse of drugs. One of the most recent examples of drug restriction is the regulated roll-out of new TB drugs (Delamanid, Bedaquiline) under the TB program. Previously, Oseltamivir phosphate and Zanamivir were made available to patients through the hospital formularies during the influenza breakout.

Few antimicrobials are in the pipeline for registration in India and are vital for treating patients with MDR pathogens. These include Plazomicin, Cefiderocol and combination drugs like Cefepime/zidebactam, Cefepime/enmetazobactum, Meropenem/vaborbactam and Imipenem/cilastatin/relebactam. While resistance to existing commonly used classes of drugs is problematic, an even more alarming trend is the rapid increase in resistance to drugs of last resort, such as carbapenems. Adding to the complexity of the issue is the disproportionate and excessive use of newer classes of antibiotics. Therefore, it becomes crucial to handle new antimicrobials responsibly. One approach is to implement stricter regulations and restrictions on the introduction of new antibiotics to the market. Such measures aim to ensure that these drugs are used judiciously and only when absolutely necessary, safeguarding their effectiveness for as long as possible. Being a clinician/intensivist/paediatrician who routinely treats sick patients having drug resistant infections, we seek your help in delineating the preferred pathways for introducing new antibiotics in the Indian market while maintaining equitable access and restricting excessive use.We request you to provide your input through the survey given below:

As per your experience, what are the drivers for irrational antimicrobial prescriptions?

*

- ◯ Diagnostic uncertainty
- ◯ Lack of availability of antibiograms
- ◯ Patient condition
- ◯ Patients demand

- Desire to preserve the doctor–patient relationship
- The doctor’s personal preferences
- All of the above
- Any other

Which sources of information do you refer to while choosing antibiotics?
*

- Local hospital guidelines
- National/international guidelines
- Journal/textbook
- Senior colleagues/ID specialists/microbiologists/clinical pharmacologists
- Internet
- Representatives from pharmaceutical companies

Do you see the need for a strong policy to regulate antibiotics meant for treating serious bacterial infections?

- Yes
- No

As newer antibiotics are lifesaving in certain situations; do you think pharmaceutical companies should be allowed to ‘market’ their products directly to prescribers?

- Yes
- No

In your opinion what level of regulation would ensure equitable access and prevent excessive use?

- Regulation at DCGI
- Regulation at hospital
- As per the judgment of the prescribing doctor
- Regulation at Unit Head level

Is there a mechanism in your hospital which helps doctors use antibiotics more rationally?

- No
- If yes, What mechanism? (select from below mentioned option)
- A. Audit and Feedback
- B. Formulary Restriction

Is the lack of a National AMR program the reason for unavailability of a suitable strategy to introduce new antimicrobials?

- Yes
- No

In your opinion will the restrictive access of new antimicrobials impact equitable access?

- Yes
- No

What could be an effective implementable way to rationalize use of new antimicrobials and minimize antimicrobial resistance?

- New antimicrobials should be introduced as Schedule H1 drugs
- New antimicrobials should be made available in hospital formularies only
- New antimicrobials should be available for compassionate use only
- New antimicrobials should be made available only after in-vitro susceptibility has been demonstrated in microbiology laboratory
- Any other suggestions
- Other:

Do you think formulary restriction will be helpful in rationalizing the use of new antimicrobials?

- Yes
- If no, what is the main challenge in formulary restriction in Indian Hospitals? (select from below mentioned option)
- Loss of clinicians' autonomy
- Lack of adequately skilled personnel to take call on use of antibiotics in sick patients

Should the new antimicrobials be available at all levels of healthcare?

- Yes
- No
- Please provide reason(s) for your answer
- Other:

Do you think there must be certain criteria for hospitals for introduction of new

antimicrobials?

- ◯ No
- ◯ If answer to the above question is yes then what should be the criteria? (select from below mentioned option)
- ◯ Accreditation of the hospital
- ◯ Hospital infection control certification
- ◯ Antimicrobial stewardship certification
- ◯ All of the above

How can we change the perception of prescribers towards a responsible antibiotic prescribing?

- ◯ Introduction of course on Antimicrobial usage in undergraduate programs
- ◯ Training of medical professionals
- ◯ Providing appropriate guidelines and protocols for antimicrobial usage
- ◯ Having a mandatory accreditation process for all doctor at fixed interval to keep the medical registration active
- ◯ All of the above

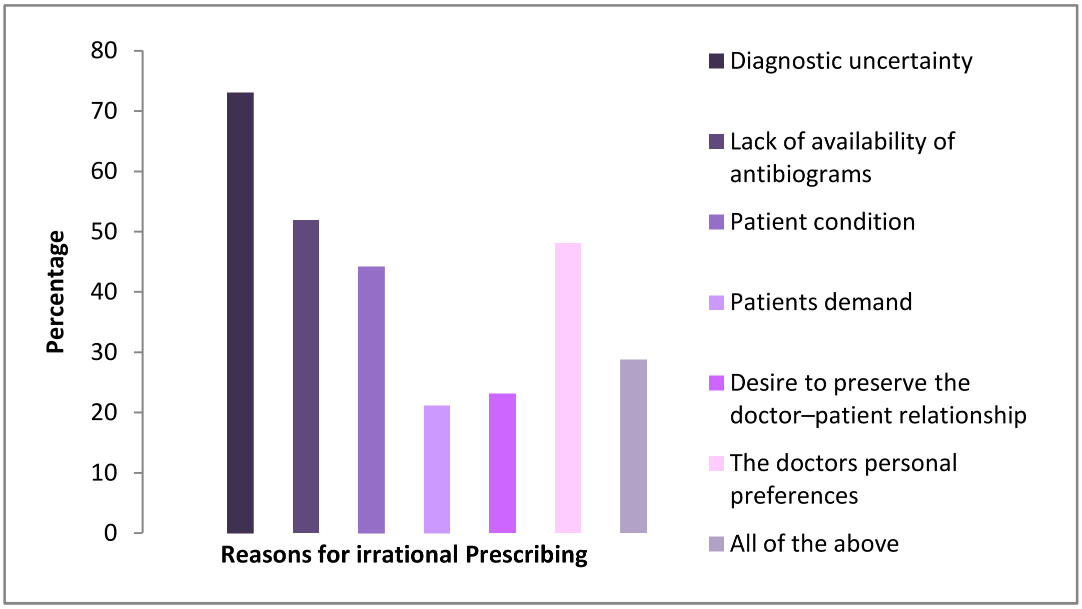

Figure S1: Reasons for irrational prescribing practices reported by physicians in India

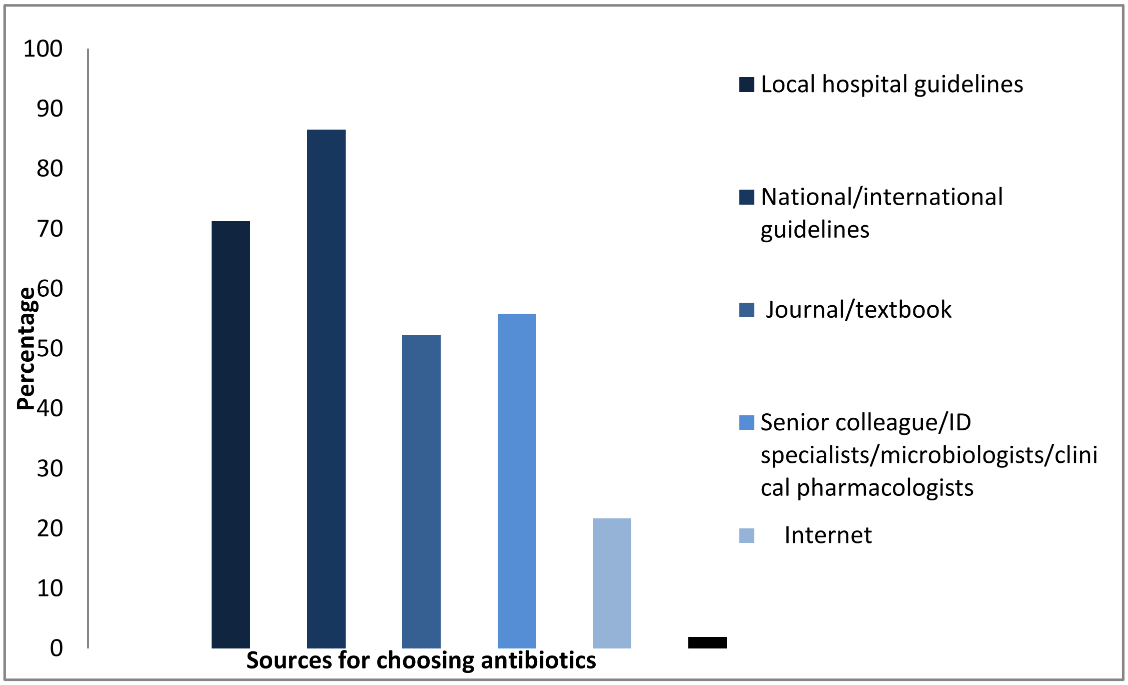

Figure S2: Sources of information referred to for choosing antibiotics by physicians in India

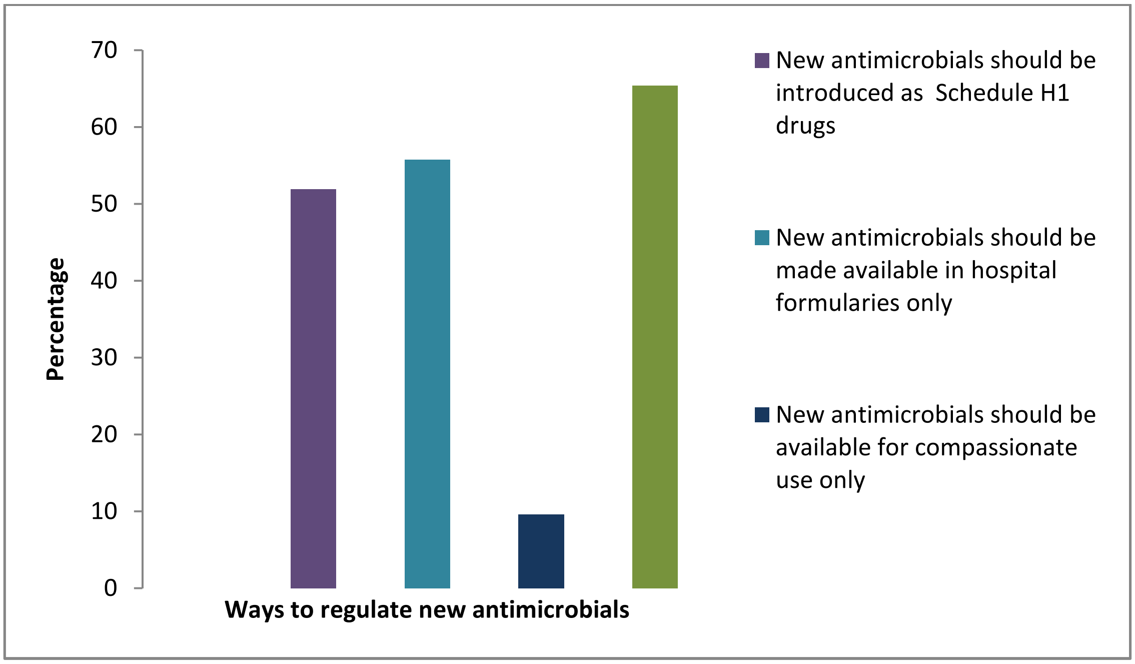

Figure S3: Mechanisms to regulate new antimicrobial use in hospital settings
